# Confined colloidal droplets dry to form circular mazes

**DOI:** 10.1073/pnas.2508363122

**Published:** 2025-08-04

**Authors:** Ilaria Beechey-Newman, Natalya Kizilova, Andreas Andersen Hennig, Eirik Grude Flekkøy, Erika Eiser

**Affiliations:** ^a^Department of Physics, PoreLab, Norwegian University of Science and Technology, Trondheim N-7491, Norway; ^b^Division of Aerodynamics, Institute of Aeronautics and Applied Mechanics, Warsaw University of Technology, Warsaw 00-665, Poland; ^c^Department of Applied Mathematics, V. N. Karazin Kharkiv National University, Kharkiv 61-022, Ukraine; ^d^Department of Physics, PoreLab, The Njord Centre, University of Oslo, Oslo N-0316O, Norway; ^e^Department of Chemistry, PoreLab, Norwegian University of Science and Technology, Trondheim N-7491, Norway; ^f^Department of Physics, University of Cambridge, Cambridge CB3 0HE, United Kingdom

**Keywords:** colloids, pattern formation, drying, self-assembly, fingering instability

## Abstract

During drying, particle-laden sessile droplets will leave so-called coffee-stain rings behind. This phenomenon is well known and well understood (Deegan *et al*., *Nature* **389**, 827–829 (1997). Here, we show that when particle-laden droplets confined in a slit are allowed to evaporate very slowly, they do not deposit coffee rings, but form surprisingly intricate, circular maze-like patterns. We present experiments that illustrate this pattern formation and discuss the factors that determine when such patterns can form. We are not aware of reports of natural examples of the formation of such beautiful patterns under confinement, although it seems likely that they exist.

## Significance

Particle laden droplets are ubiquitous. When such droplets dry, they deposit the suspended particles in nonuniform patterns. An example is sessile droplets of coffee, which, upon drying, leave characteristic circular “coffee stains.” The formation of circular coffee stains is due to the combined effect of evaporation, flow, and pinning of the droplet’s circumference. Here, we show that the slow drying of confined droplets proceeds very differently and may result in a surprisingly intricate (and beautiful) labyrinthine pattern. The mechanism underlying this drying process is rather different from that behind the formation of circular coffee stains. Our findings are likely to be of wide relevance, as the controlled drying of particle laden droplets is common and of practical importance.

Patterns exist everywhere in the natural world. Striking examples are Romanesco broccoli (1), snail shells, or the arrangements of sunflower seeds (2). Similarly, animals exhibit a multitude of striking geometrical patterns, and the human brain has evolved to recognize such patterns (3). Of course, we do not only enjoy observing intricate natural patterns, we also want to understand the physical mechanisms by which they form. An early example of the physics behind pattern formation is the work of Turing on the patterns generated by reaction–diffusion processes (4, 5).

However, patterns need not be intricate to be interesting from a physics perspective. A case in point is the mundane “coffee-stain” effect that is observed when a sessile droplet containing a particulate dispersion (e.g. coffee) dries on a flat, partially wetting surface. The formation of the resulting circular coffee stain was studied and explained in a number of papers by Deegan et al. (6, 7). These papers argue that the accumulation of a ring of particles is primarily driven by the hydrodynamic flows caused by the high rate of evaporation at the rim of the sessile droplet. For many practical applications, such as ink-jet printing (8), it is important to control the shape and uniformity of the solid deposits, which depend strongly on the size of the drops and their evaporation rate (9). Depending on the circumstances, drying sessile droplets may form single or multiple circular rings, uniform deposits, or simple patterns and even short radial fingers in the case of a combined capillary and Marangoni flow (10). Yet, they typically do not exhibit intricate patterns, even when confined between two parallel plates, but bounded by an air–water interface on the circumference (11–14).

**Fig. 1.**
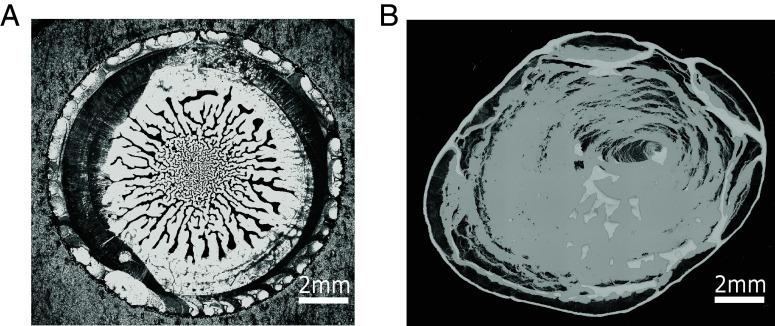
Drying pattern produced by a confined droplet of an aqueous suspension containing 1 wt% of charge-stabilized colloids with a diameter of 1.8 μm, compared to an equivalent, unconfined droplet. The bright-field microscopy images show (*A*) the sediment deposited by a slowly dried droplet in cylindrical confinement, and (*B*) the sediment deposited by a sessile droplet that has evaporated in dry air.

Here, we show that vertical confinement of drying, particle-laden droplets can lead to very different (and aesthetically pleasing) patterns. In particular, we consider the practically important case of cylindrical, particle-laden droplets bridging the top and bottom of the confining slit, under conditions where the evaporation rate is throttled to be very low. Under these conditions, the shrinking droplet undergoes a sequence of instabilities resulting in the formation of a spherical “maze,” with a pattern that gets finer toward the center (Fig. 1).

As we argue below, this pattern formation is possible under conditions where the rim of the droplet is not pinned to the surface. At some point, the accumulation of colloids advected to the side walls of the cylindrical droplet causes the effective surface tension to change sign, leading to a fingering instability. The tips of these fingers are arrested, but the invaginations in between (the “air fingers”) can grow, and split, leading to ever finer channels.

## 1. Experiments

### 1.1. Drying Process.

**Fig. 2.**
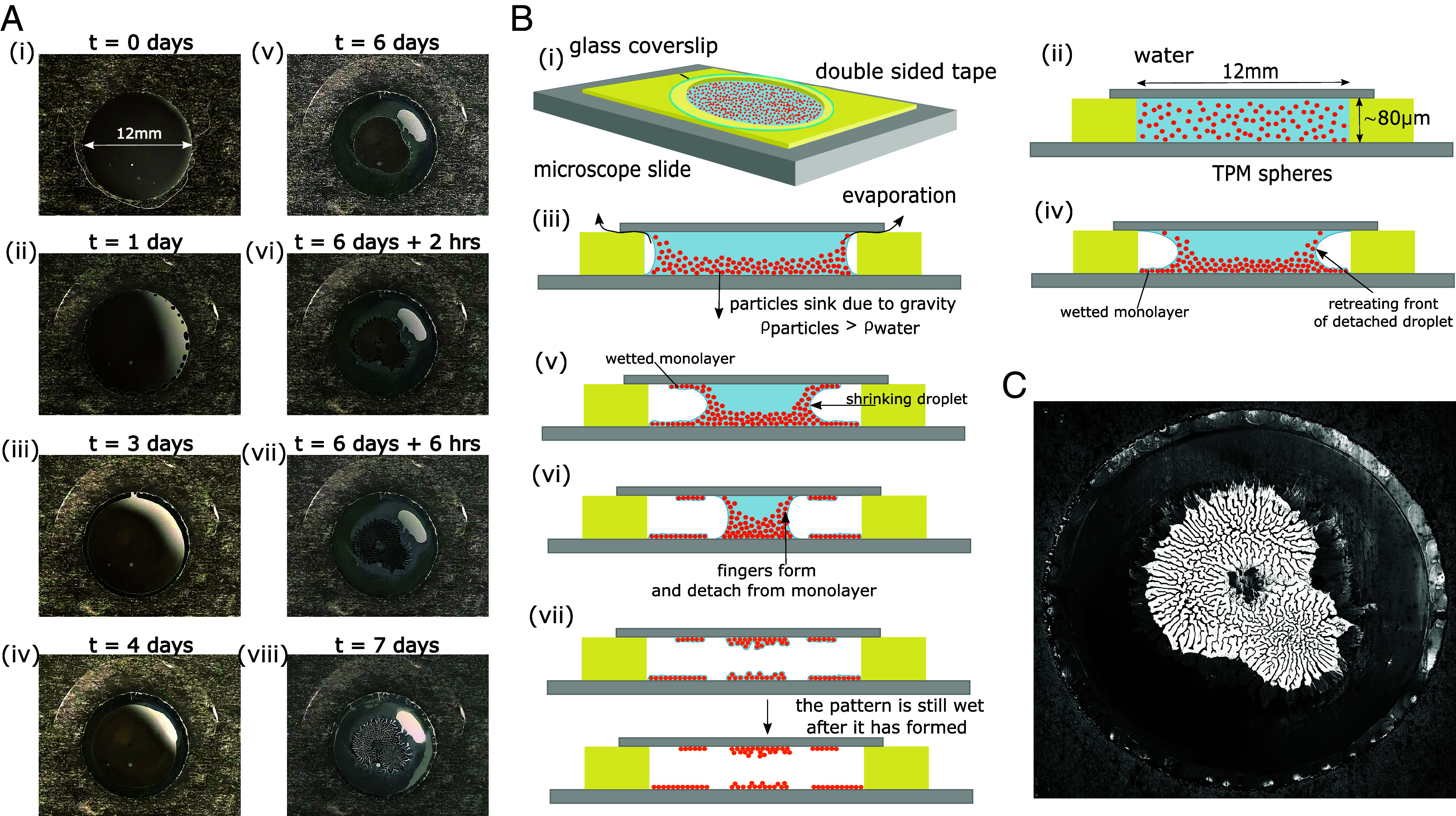
Different stages of a drying droplet confined and quasi-sealed between two glass surfaces. (*A*) Photographs of the sample from time-lapse Movie S1, taken during 7 d of drying. (*B*) Schematic of (*i*) the sample geometry, (*ii*) a cross-section of the sample at t=0, (*iii*) the bubble formation due to evaporation of water across the gap between the sticky tape and the coverslip, (*iv*) the droplet detachment from the edge and the beginning of a monolayer deposition on the *Bottom* surface. (*v*) Subsequently, the sedimented colloids are pushed up the air–water interface, driving a monolayer deposition on the coverslip as well. (*vi*) The monolayer deposition stops abruptly, followed by finger formation, through which the fingers remain connected between the *Top* and *Bottom* surfaces. (*vii*) The final pattern of fingers is formed and the liquid bridge between the surfaces breaks: Similar colloidal patterns remain on both surfaces but are still wet. (*c*) Microscopy image of the fully dried sample.

Fig. 2*A* shows a sequence of photographs (taken from Movie S1) of an evaporating cylindrical droplet containing a dispersion of spherical TPM colloids (diameter 1.8 μm–see *Materials and Methods*), bounded by glass surfaces on top and bottom, and confined in a quasi-sealed cylindrical cavity. Fig. 2*B* shows a cross-sectional schematic drawing of the droplet evolution, and Fig. 2*C* shows the pattern that remains once the droplet has dried.

The cylindrical cavity containing the dispersion was created by punching a hole of 12 mm in diameter in a piece of double-sided sticky tape that was pressed onto a clean microscope slide. After placing ∼30 μL of a 1 wt% colloidal suspension in the cylindrical cavity, a circular coverslip was carefully placed on top of it. This procedure resulted in a slight overfilling of the cylindrical cell, which had a height of ≈80 μm. The coverslip was not firmly pressed onto the sticky sealing tape, thus allowing for very slow evaporation of water vapor out of the cell and slow permeation of air into the cell (*SI Appendix*, Fig. S1 and Movie S2).

Typical drying times were 8±2 d: If the evaporation had not been throttled, the same droplet would evaporate in a matter of minutes. This point is important because it implies that the escape of water vapor through the cell walls is the rate-limiting step in the evaporation. This observation implies that the water vapor pressure throughout the cylindrical cell is very nearly constant at a value that is just a few percent below the vapor pressure of water at the ambient temperature and that the evaporative mass flux of water is effectively constant, except at the very end of the drying process. The evaporation of the droplet was observed to proceed in three distinct stages. In the first stage (stage 1), starting at the moment of the sample preparation, we observed the slow formation and growth of air bubbles at the edge of the cell (Fig. 2*A*, *ii*). After about 4 d, these bubbles had grown enough to merge to form an annulus, enclosing a cylindrical droplet of the TPM suspension. The sketches in Fig. 2*B*, *ii*–*iv*) present a cross-sectional view of this capillary bridge. Occasionally, we observed that the initially even distribution of colloids, seen as homogeneous whitish scattering light, photographed in reflection, becomes skewed (see Fig. 2*A*, where more colloids can be seen in the *Top Right* of the sample). The formation of such off-center colloidal patches only happens if the sample is not held perfectly horizontal: After the TPM particles sediment, which typically happens within hours, the sedimented colloids can still diffuse, causing the sediment to accumulate preferentially on one side of the cell. This asymmetric accumulation of colloids in the droplet does not change the qualitative features of the subsequent evolution of the drying pattern.

Interestingly, microscopy experiments show that when the detached droplet shrinks (stage 2), a colloidal monolayer is deposited on the bottom surface, and, to a lesser extent, on the top surface. We note that the barometric height of the TPM colloids, $h_{B}=\Delta \;{\text{mg/k}}_{\text{B}}\text{T}$, is only slightly smaller than the colloidal diameter ($k_{B}$ is Boltzmann’s constant, T the absolute temperature, Δm the mass difference between the colloid and displaced water, and g the gravitational acceleration). The deposition of colloids on the top surface is therefore surprising, as the cell height is some 50 times larger than $h_{B}$. The reason that the colloids can reach the top surface is that the evaporation-driven hydrodynamic flow to the droplet–air interface is strong enough (high enough Péclet number) to cause a flow-driven colloidal pressure near the interface that is large enough to cause the accumulation of the layer to expand in the vertical direction. In the sample shown in Fig. 2*A*, the colloidal monolayer deposition on the *Bottom* and *Top* surfaces of the cell took place from day 4 to day 6. Colloidal monolayer deposition by the retracting contact line of a colloid-laden droplet has been well studied (15, 16) and has been exploited to grow defect-free crystalline colloidal layers for photonic applications (17). There is a sample-to-sample variation in the density of the deposited colloidal layers, but they always appear after the droplet has detached from the walls of the cell. Microscopy imaging of fluorescently labeled TPM particles (*SI Appendix*, Fig. S2 and Movie S3) confirms the deposition of the TPM colloids on both the bottom and the top surfaces.

The width of the accumulation region of TPM particles advected to the air–water interface is 20 to 30 μm, which rules out that this layer is due to the “Pickering” mechanism where colloidal particles are embedded *in* the interface, as seen for particle-laden oil-in-water droplets (18, 19).

**Fig. 3.**
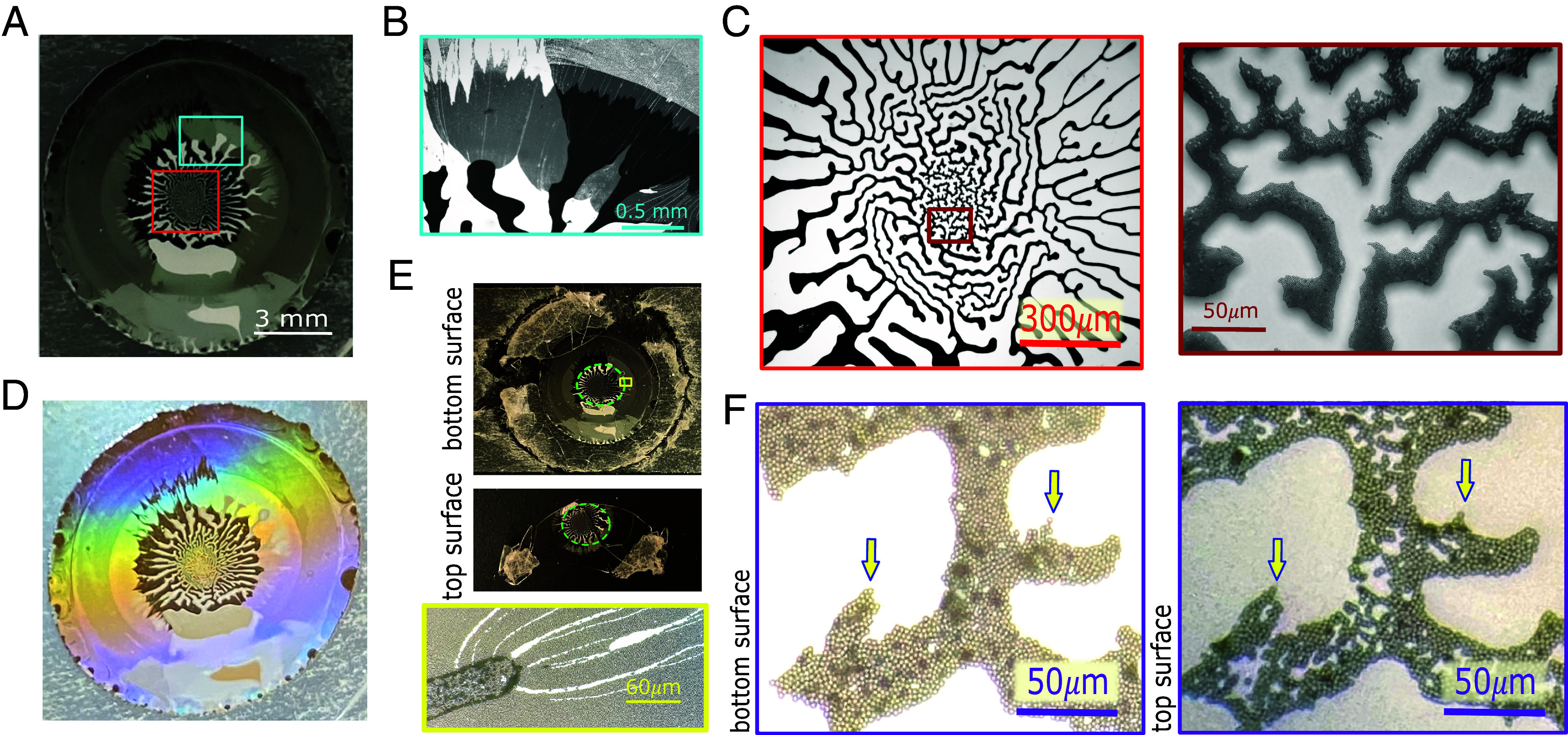
Photographs and bright-field microscopy images of a fully dried sample. (*A*) Photograph taken in reflection. Bright-field microscope images obtained of the (*B*) cyan and (*C*) red encased regions shown in (*A*), taken in transmission. (*B*) shows the monolayer-to-finger transition, while the images in (*C*) illustrate the abrupt change in length scales and orientations of the fingers, taken with increasing magnification. (*D*) Photograph of the sample taken in transmission, using the white light source of the microscope. (*E*) Photographs of the opened-up sample show similar dried patterns on the *Bottom* and *Top* slide (green-dashed circles), while the yellow rectangle is a zoom-in on one of the monolayer-to-finger transition regions on the *Bottom* surface. (*F*) High magnification micrographs of the mirror-imaged colloidal arrangements in the center of the sample. The arrows show mirrored features.

Stage 3 is marked by a dramatic change in the evolution of the drying droplet: The monolayer deposition stops abruptly, and the roughly cylindrical symmetry of the droplet is broken, as can be seen in Fig. 2 *A*, *vi*–*viii*). These figures show the formation of fine, white “fingers” containing the colloids and black, colloid-free regions outside the shrinking droplet. This finger formation is rapid in comparison with the first two stages and is completed after around 4 to 5 h in this sample. However, the drying process is not yet finished at this point, as the “white” fingers containing the colloids are still wet, and colloidal motion can be observed inside them—even at the interface. Moreover, the droplet still connects the *Top* and *Bottom* surfaces of the cell as depicted in Figs. 2 *B*, *vi* and 4*D*. Confocal microscopy images, shown in *SI Appendix*, Fig. S2, demonstrate that the entire labyrinth structure and the deposited monolayers appear to be still wet after the fingers have formed. The water bridge between the top and bottom only ruptures after t=7 d. After the liquid bridge between the top and bottom had ruptured, we could open up the cell and image the patterns left on the opposing surfaces (Fig. 3 *E* and *F*). These images show that the pattern on the top surface and the mirror image of the bottom pattern are indeed very similar, as is to be expected if they resulted from the drying of the same droplet.

**Fig. 4.**
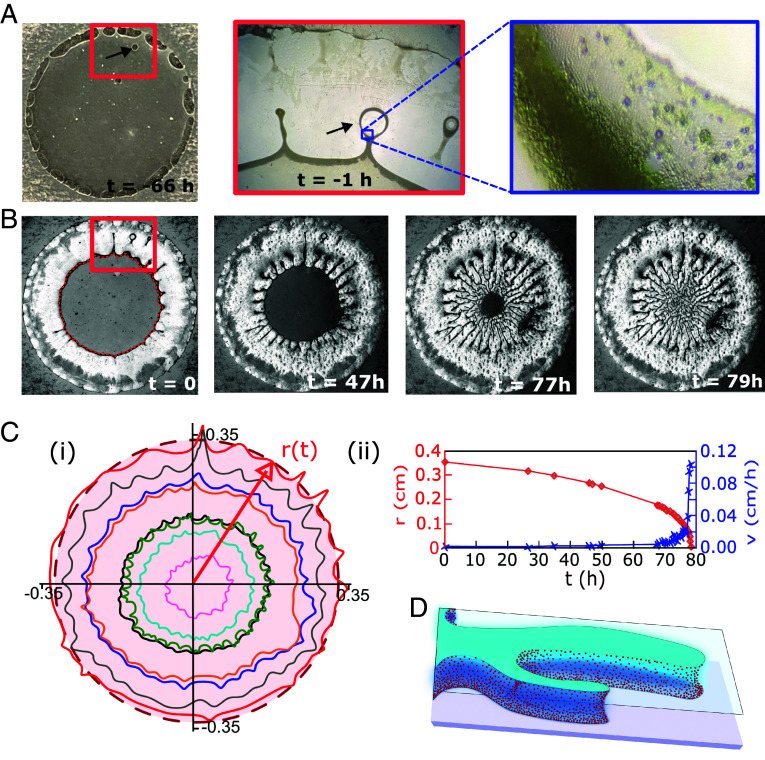
Drying kinetics of a confined droplet containing 1 wt% of fluorescently labeled, 1.8 μm large TPM particles. (*A*) Photograph of the sample, taken 72 h after preparation and 66 h before starting the time-lapse Movie S4. The photo and microscope images (red and blue squares) were taken 1 h before starting the V4 recording and capture the onset of finger formation. The black arrows show an air bubble that is trapped by the accumulation of colloids at the water–air interface. Other air bubbles escape to the outer rim of the sample. (*B*) Snapshots from the time-lapse series shown in Movie S4, starting at the relative time stamp t=0. The sequence of photos shows the progressive shrinkage of the inscribed circle with average radius r(t), excluding the fingers. (*C*, *i*) Traces of the drop interface inscribing the area inside the black appearing rim of the droplets as a function of time. The outer red trace is taken for the photo taken at t=0. (*ii*) Plot of the change of r(t) (red) and velocity v(t) (blue) of the inscribed circle. The solid lines are guides to the eye. (*D*) Illustration of individual fingers; they remain connected between the *Bottom* and *Top* surface until the entire pattern is formed.

Several factors may distort the radial symmetry of the drying patterns. For instance, trapped bubbles (as in the sample shown in Fig. 2) may distort the pattern, and, as previously discussed, even a slight lack of horizontal alignment can distort the pattern. A gallery of drying patterns of analogous samples is presented in *SI Appendix*, Fig. S4, demonstrating the reproducibility of this process. Movie S1 shows a time-lapse movie of the drying process depicted in Fig. 2.

During stage 3 there is a sudden change in the length scale and/or orientation of the fingers, as is demonstrated in Fig. 3. In that figure, we present another fully dried sample photographed both in reflection (Fig. 3*A*) and transmission mode. The latter was taken using the white-light source of the microscope: The iridescent colors in Fig. 3*D* stem from the colloidal monolayers that diffract the transmitted light. Fig. 3*B* focuses on the transition from the monolayers to fingers, while Fig. 3*C* focuses on the central finger-pattern and a further zoomed-in image of the finer structure at the center, where we can see individual colloids.

The yellow-framed microscope image zooms in on a finger in contact with the monolayer on the bottom surface. The white streaks in the monolayer that seem to radiate from the fingers are cracks that appear in the final drying stage. These cracks are associated with an instability caused by thermal fluctuations in thin films and a subsequent volume reduction during the final drying and have been observed in various systems (14, 20, 21).

### 1.2. Interpretation.

As is clear from the above discussion, the pattern formation that we observe depends on the synergy of several factors: The droplet must be confined, it must evaporate very slowly, the contact lines (top and bottom) should not be pinned (at least not initially), and the diffusion of the colloids should be fast enough to inhibit the formation of a “coffee ring” that would pin the radius of the droplet. These are just some of the factors that have to be “just right” (others include the nature of the solvent and the shape and concentration of the colloids). Below, we provide a tentative explanation of how the different factors align to generate the intricate drying patterns that we observe, as presented in Fig. 3.

The drying kinetics of a sample with radial symmetry is shown for different stages of pattern formation in Fig. 4. The images in Fig. 4*B* are taken from the time-lapse Movie S4.

#### 1.2.1. Early stage.

The cylindrical cell that contains the suspension droplet has been filled, but not hermetically sealed. Consequently, water evaporates and air flows in slowly from the outer rim of the cylindrical cell. A visible manifestation of this throttled gas exchange is the slow growth of air bubbles along the outer rim of the cylindrical cell. These grow and merge to form a roughly circular air–vapor droplet boundary (capillary bridge).

In the experiments, this detached droplet dries in about 5 d from an initial radius of ∼5 mm. The average observed drying speed is then of order 10^−6^ cm/s, which is much slower than the estimated shrinking speed that the same droplet would have if it were in contact with dry air [∼2.7 10^−5^ cm s^−1^ (*SI Appendix*)]. The fact that the observed shrinking speed is about thirty times slower than in dry air indicates that the relative humidity (RH) of the air outside the droplet is close to one. In fact, as

$${\dot{r}}_{\mathrm{partial}}=\left(1-RH\right){\dot{r}}_{\mathrm{sat}},$$

we estimate that the air around the water droplet is approximately >96% saturated. At this relative humidity, water will form a very thin “wetting” film on the glass (silica) surface outside the droplet. Under these conditions, the height of the wetting layer is in the nanometer range, which is much smaller than the size of the TPM particles. Hence, colloids will not enter this film spontaneously. However, there will be a flow through these thin films, dominated by the flux through their rims, which is well documented (16, 22, 23). The resulting high fluid-flow velocity (Péclet number appreciably larger than one) drags colloids from the droplet into the thin film (i.e. the films are connected to the central droplet; see *SI Appendix*, Fig. S2).

Initially, the droplet is roughly cylindrical. If the evaporation rate is controlled by the permeability of the tape bounding the cell, the amount of mass M evaporating per unit time ρdV/dt is constant; ρ is the density of water and $V=\pi r^{2}\left(t\right)h$ is the volume of the cylindrical droplet. The constant rate of water evaporation implies that, as long as the droplet remains cylindrical, the rate of change of the area of the droplet is $dr^{2}/dt=\text{constant}$. Therefore, $r^{2}\left(t\right)=r_{0}^{2}-\alpha t$, where α is a system-dependent constant. The experimental data for the initial droplet shrinkage agree well with the above function, even though we observe the deposition of a colloidal monolayer (stage 2, lasting from t∼−66 h until about t∼−1 h; see Fig. 4*B* and *SI Appendix*, Fig. S3–here we define t=0 as the moment shortly after the finger formation, stage 3, starts). After the fingering instability appears, we can still define the location of the shrinking front, but the effective radius of this front moves faster than the front of a shrinking cylindrical droplet.

Due to the evaporative flux through the side of the droplet, charged TPM colloids will be advected to this interface (or, more precisely, the interface “sweeps up” colloids). But the colloids can also diffuse. The competition between the speed of advection ($v\approx 10^{-6}$cm s^−1^) and the self-diffusion coefficients of TPM colloids in water(D = $O(10^{-8})$ cm^2^ s^−1^) results in the formation of a fluid colloidal “crust” terminated by the air–water interface. In steady state, when the number of colloids deposited balances the number of colloids advected, the typical thickness of this crust is D/v, which is of the order of dozens of colloidal diameters. The above estimate yields just an order of magnitude because the “sweeping up” of the colloids mainly takes place at the bottom of the cell: The colloids are then moved up due to the osmotic pressure of the dense suspension at the air–water interface, and diffuse away into the bulk of the droplet, where they sediment again. However, a fraction of the colloids are swept along the air–water interface to the top rim of the droplet, where they are deposited in a monolayer on the top surface of the cell (Fig. 2 *B*, *v*).

#### 1.2.2. Monolayer to finger transition.

As the central droplet shrinks, three things happen: 1) The area of the external, colloid-supported wetting film grows and carries a growing fraction of the evaporative flux (15). 2) The radial shrinkage speed of the droplet increases, as explained above. 3) There is an accumulation of colloids near the rim of the droplets.

The result of these effects is that, at some point, the transport of fluid to the colloid-supported film cannot keep up with the rate of evaporation of this film. When this happens, the film breaks, and the evaporative flux through the rims of the droplet drops sharply, causing the colloid deposition outside the droplet to stop, as seen in the experiments. From then on, the droplet shrinks mainly due to evaporation through its vertical surfaces (stage 3).

#### 1.2.3. Surface instability.

As the suspension inside the droplet is fluid, colloids are still being transported to the liquid–vapor interface by the evaporative flow. However, as the colloids are now no longer deposited in the wetting film, they accumulate on the vertical surface of the droplet, thickening the colloidal “crust” at the air–water interface (*SI Appendix*, Fig. S2). The flow of solvent causes an additional stress in the crust. As the crust is fluid, it is plausible that this stress is isotropic and has therefore a transverse component that counteracts the surface tension of the air–water interface. At some point, this transverse stress overcomes the air–water surface tension, and the interface starts to crumple (a description of the buckling of “solid” particle crusts is explained in refs. 24 and 25).

Importantly, as the overall surface tension of the droplet is now negative, the Laplace pressure of the tips of the fingers is negative, while it is positive at the points of maximum indentation (26). The tips of the fingers will not move, because such motion would increase, rather than decrease the volume of liquid in the fingers. However, the water transport continues to feed the evaporation through the surface of the fingers. Evaporation of the water in the fingers compacts the colloidal suspension in the fingers, to the extent that they undergo structural arrest. Indeed, we observe in the experiments that fingers, once formed, retain their shape.

However, the tips of the air fingers can move inward, as they face the much more dilute colloidal suspension in the remaining central part of the droplet. As more water evaporates, the colloidal concentration in the central part of the droplet continues to increase, and at some point, the Laplace pressure of the invaginations is insufficient to move the surface. At that point, the droplet undergoes a secondary fingering instability, such that the curvature at the tip of the air fingers can overcome the colloidal osmotic pressure. Indeed, in the experiments, we observe a fairly sudden splitting of the air fingers. In principle, this scenario could be repeated, but in our experiments, it is observed only once, before the colloidal suspension inside the fingers undergoes structural arrest.

## 2. Conclusion

The experiments presented here demonstrate that the slow evaporation of a confined, particle-laden droplet can be qualitatively different from the patterns that form during the drying of sessile droplets.

We note that it is the confinement that is the crucial factor in the emergence of the fingered patterns that we observe. The fact that the droplet is cylindrical is not essential. In fact, similar patterns form when studying the drying of a colloidal suspension in a rectangular cell (*SI Appendix*, Fig. S5).

As multilayer structures are common in natural materials, it seems likely that the kind of patterns that we observe in confined cells, also occur in naturally occurring materials.

## 3. Materials and Methods

### 3.1. Chemicals.

All chemicals purchased were used as received. 3-(trimethoxysilyl)propyl methacrylate (TPM) oil (98%, Sigma Aldrich), ammonium hydroxide solution (28% NH3 in H20, 99.99%, Sigma Aldrich), 2,2-Azobis(2-methylpropionitrile) (AIBN 98%, Sigma-Aldrich), Emprove Expert 4M hydrochloric acid (HCl, Sigma Aldrich), DMSO (ACS reagent >99%, Sigma Aldrich), THF, BODIPY 505/515 (Thermo Fischer Scientific), Chloroform (anhydrous, 99%, Sigma Aldrich), 3-aminopropyl trimethoxysilane (97% Sigma Aldrich), Pluronic F-127 (Sigma Aldrich), and deionized water from Merck Millipore Milli-Q Direct-Q 3 UV purification system.

### 3.2. Synthesis.

We synthesized TPM (3-(trimethoxysilyl)propyl methacrylate) particles of diameter $d_{p}∼1.8$ μm, following a simple bench-top synthesis route (19). For this we measured 100 mL deionized water in a 250 mL beaker. While stirring the water on a magnetic stirrer (400 rpm) 100 μL of 28 wt.% ammonia was added, followed by the addition of 1 mL TPM oil. We then allow an emulsion to form for 1 h and 15 min. Subsequently, we added 10 mg of AIBN (azobis(isobutyronitrile)), a radical initiator. After a couple of minutes, the mixture was transferred to a flask and sealed with a screw top, and heated at 80 ^°^C for 3 h. The resulting colloidal dispersion was washed with deionized water in 3 centrifuging and decanting cycles. The zeta potential of these particles is reported to be about −36 mV at pH 5.6, their density is 1.314 g cm^−3^, and their refractive index is 1.512 (19).

For use in confocal microscopy, we used a different synthesis so as to incorporate a fluorescent dye into the particles (27). This method produced particles with a slightly smaller diameter of ∼1.3 μm. To do this, we first added 2 mg of BODIPY to 2 ml of chloroform with 2μl of 3-aminopropyl ethoxysilane and leave it to react for 24 h. The following day we started by mixing 20 ml of 0.5 mM HCl solution with 2 ml TPM oil for 1 h until the mixture was no longer cloudy. To make 1.3 μm diameter particles, we mixed 6 ml of the resulting solution with 19 ml of 0.5 mM HCl solution in a 100 ml round bottom flask for 10 min (600 rpm). Next, we added 25 ml of 0.028% ammonia solution and let it sit for 45 min. Then 400 μl of the BODIPY solution was added and mixed for 1 h, and following this 10 mg of AIBN was added and the mixture was left to stir for another 30 min. Finally, the mixture was placed in an oven at 80 ^°^C and tumbled every 30 min for 3 h. The resulting suspension was cleaned in the same way as before.

In either case, the final TPM suspension was kept for later use at 4 ^°^C.

### 3.3. Bright- and Dark-Field Microscopy and Photography.

Bright-field and dark-field microscopy were performed using an Amac microscope, equipped with 5×, 10×, and 40× objectives, and a CMOS camera. Larger fields of view were photographed with an i-phone 13 through the eye-piece with 10× magnification. Timelapse movies were recorded with a FUJIFILM X100F digital camera with a Fujinon aspherical lens, Super EBC, f=23 mm 1:2, from Japan.

### 3.4. Confocal Microscopy.

The TPM particles were fluorescently labeled, either during the synthesis process, as described before, or by following a swelling–deswelling procedure, after the basic synthesis, introduced by Oh et al. (28) and described for polystyrene particles by Zupkauskas et al. (29): We mixed 10 mL of a 1 wt% TPM suspension with 20 mL of THF (tetrahydrofuran). Subsequently, 200 μL of BODIPY in DMSO (1 mg/ml), and the mixture was rigorously stirred for 30 min. Then an excess of 200 mL DI-water, heated at 60 ^°^C for 20 min, and the washed 3× by centrifugation. A Leica DMi8 SP8 confocal microscopy was used to image the drying of the confined TPM suspension.

## Supplementary Material

Appendix 01 (PDF)

Movie S1. V1 - Time-lapse movie of drying process for sample presented in Figure 2a in the main text.

Movie S2. V2 - Time-lapse movie of finger formation process of sample presented in Figure S1.

Movie S3. V3 - Full stack of confocal images taken at the droplet interface presented in Figure S2.

Movie S4. V4 - Time-lapse movie of finger formation process for the analysis presented in Figure 4 in the main text, and Figure S3.

## Data Availability

All raw image data, including confocal microscopy .lif files, has been deposited in Zenodo (DOI: 10.5281/zenodo.14881850) (30). Images have been deposited in Zenodo (DOI: 10.5281/zenodo.14881849) (30).
