# Supplementary material for: Confined colloidal droplets dry to form circular mazes
Source: Proc Natl Acad Sci U S A. 2025 Aug 4;122(32):e2508363122. doi: 10.1073/pnas.2508363122 (PMC12358886; doi:10.1073/pnas.2508363122)
Supplement: Supplementary file 1 — Appendix 01 (PDF) [file pnas.2508363122.sapp.pdf]

Supporting Information for

Confined colloidal droplets dry to form circular mazes

Ilaria Beechey-Newman, Natalya Kizilova, Andreas Andersen Hennig, Eirik Grude Flekkøy, and Erika Eiser

Erika Eiser.
E-mail: erika.eiser@ntnu.no

This PDF file includes:

Supporting text
Figs. S1 to S5
Legends for Movies S1 to S4
SI References

Other supporting materials for this manuscript include the following:

Movies S1 to S4

Supporting Information Text

Fig. S1 **a**(i) illustrates schematically (with a top-down and cross-sectional view) how the water slowly evaporates through the gap between the coverslip and the double sided tape that serves as spacer and confining walls of the flat sample cell containing 1 wt% of 1.8 μm large TPM particles. Here, the water was fluorescently labelled with the green fluorescent dye calcein (excitation at 495 nm, emission at 515 nm) in order to visualize the water in the sample. Fluorescent images (512×512 pixels, with pixel size 4.55 μm) were taken with a 5× objective lens, while focused in the middle of the cell (approximately 40 μm from the bottom surface), and with a long exposure time to visualise where, and how much, of the fluorescent water is present. In all images, blue indicates a high water content (saturating the monitor for the green dye), red indicates a much lower water content and yellow an intermediate one.

Fig. S1 **a**(ii) shows a zoomed in image taken at the edge of the cell that demonstrates that water and air can be transported between the coverslip and the double sided tape. Fig. S1 **a**(iii) is a 5×5 grid of images taken, as described above, to generate a picture of the entire sample at the moment finger formation starts. We refer to this drying period as **stage 3** in the main text. This stage signifies the moment when the monolayer deposition on top and bottom surface stops and fingers start to form. The yellow to red coloring in the center of the droplet reflects the depression of the coverslip also depicted schematically in the sample cross-section in **a**(i) and **b**(i). This depression appears within the first 6 hours of evaporation and remains until the entire fingering pattern has formed. This is due to the capillary force produced by the capillary bridge (1). Assuming a wetting angle θ of roughly 45°, an average height $h = 60\,\mu$m and radius of $r =$ 5mm, we estimate the capillary force pushing the two surfaces together to be of the order of 0.15 N, which is sufficient to deform the coverslip while the double sided tape represents the counteracting force.

Fig. S1 **b**(ii) is a tiling of confocal image stacks taken across the sample thickness (50 images taken over a sample thickness of 80 μm), shortly before the onset of finger formation. The entire strip is 7 mm long and 0.6 mm wide, starting from the middle of the sample (bottom) and ending at the tape at the sample edge (top). The drop of fluorescent intensity towards the center of the droplet (decreasing green levels) shows the depression of the sample due to capillary forces in the middle.

Fig. S1 **c** shows the tiling of confocal-images of the entire sample, in exactly the same way as for **a**(iii), but just after the fingering pattern had finished forming. The blue, yellow and red show that the fingers and the monolayers are still wet, but that the monolayers and central fingers dry first while the finger tips dry last. The entire sequence of the finger formation is shown in Supporting Video **V2**, in which the individual images were taken in 20 minute intervals, over the course of 4 hours and 20 minutes.

In Fig. S2 **a** we illustrate schematically both a top-down and cross-sectional view of a small droplet of the colloidal suspension placed in the middle of the sample cell, such that it does not reach the wall of the cell made by the double sided tape. This allowed us to observe the interface between the vapor phase and the droplet confined between the top and bottom surface in a controlled way. Here we used 1.3 μm large TPM particles that were fluorescently labelled with BODIPY (2).

Fig. S2 **b** shows two confocal microscopy images taken across a 200 μm wide section at the air-droplet interface (blue dashed rectangle in the top view); they were recorded at the bottom ($z = 0$) and near the top ($z = 65\,\mu$m) of the sample and are part of a z-stack of images leafing through the entire sample thickness, which is shown in Supporting Video **V3**. In Fig. S2 **c** we plot the total intensity obtained for each z-layer of 2 μm thickness and averaged in y-direction that are shown in **V3**. The grey-scales in Fig. S2 **b** indicate the intensity of the fluorescence coming form the colloids: higher particle concentrations show stronger scattering intensity and thus a darker gray. The outside of the droplet is white as it is completely void of particles. At $z = 0\,\mu$m, we see the onset of a colloidal monolayer deposition at around $x \approx 340\,\mu$m followed by a sharp intensity increase at $x \approx 310\,\mu$m (thick dark line) that coincides with the onset of the steep vapour-droplet interface. At even smaller x-values the scattering intensity levels off, reflecting a constant bulk concentration of the fluorescently labelled colloids inside the droplet. From the intensity curves across the vapor-droplet interface we estimated the mean-width half-value (MWHV) as function of z. We found a MWHV of roughly 25 μm for all z-values. However, the colloid density of the particles at the bottom is higher than at the top.

In Fig. S2 **c** the intensity curves obtained from integrating the intensity (gray scale) in the xy-plane are plotted as function of z in a staggered way so that the intensity at the top-surface appears at lower intensity values and the bottom surface is shown at higher intensity values. In the inset we plot the intensity values as function of z for each intensity curve, summed up from $x = 0$ to 225 μm). The dashed line is an exponential fit to the intensity curve showing that near the air-water interface the colloids are Boltzmann distributed in z.

In Fig. S3 we present different ways of analysing the shrinking area of the confined droplet as function of time, which is presented in Fig. 3 in the main text and video **V4**. Once finger deposition starts, the progressively shrinking drop area A, as seen from the top, can be described either by the total area of the fingers plus the inscribed circle or by the inscribed circle only. We developed a Python code to measure these two areas from the time-lapse video **V4**.

In the analysis, we make use of two different methods of calculating the inscribed circle. Method A1 is based on applying a Sobel filter on the time-lapse images to find the lines of maximum contrast in the images. Then, we apply a Gaussian blur to be able to distinguish the centre from the fingers. We perform an image threshold based on Otsu's algorithm to binarise the images, and then we perform image segmentation based on the Hoshen-Kopelman algorithm to separate all colloid-rich (i.e. whiter) areas of the images. Subsequently, we count the number of pixels in the central cluster, which is the total area of the inscribed circle A_1. We analyse the radius of this circle, which we denote $r_1(t) = r_1$.

Fig. S3 also contain two smooth curves, which are based on the scaling relation

$$\frac{dr_1}{dt} \sim 1/r_1^2 \iff \frac{dA_1}{dt} \sim 1/r_1 \qquad [1]$$

Assuming that $r_1 \to 0$ at the end of the drying process, we find the proportionality constant in eq. Eq. (1) to be

$$C = -\frac{1}{3\Delta t} r_{1,0}^3 \ ,$$

where $r_{1,0} \approx 3.5$ mm is the radius of the inscribed circle when we start to observe the fingering instability, and Δt is the elapsed time of the instability (i.e. from $t = 0$ in Fig. S3).

In the second method, we assessed the area $A_2(t)$ using the WebPlotDigitizer from Automeris LLC. In this method we estimated a slightly larger area than for method 1, as it considered the outer perimeter of the inscribed circle but excluded the finger. We could fit the resulting area $A_2(t)$ with the same functional form with a larger constant ($C = 0.59$).

We observe that this scaling relation only applies *after* the fingering instability occurs, as seen by the extrapolated dashed lines in Fig. S3. Thus, we have a transition from an expected linear scaling $r^2(t) = r_0^2 - \alpha t$ with α as the system-dependent drying rate, to the scaling relation proposed for the fingering region.

In the first row of Fig. S4, we show the final drying patterns of several other samples, made in the same way as those shown in the main text, to demonstrate the highly reproducible nature of these patterns. In the second row, we then show 4 more samples, each prepared under slightly different conditions. Typically, using lower concentrations of TPM particles lead to similar drying patterns, but with a less dense monolayer deposition than the samples containing 1 wt% TPM particles. In contrast, using higher concentrations of TPM particles lead to similar monolayer deposition behaviour as for the samples containing 1 wt% TPM particles, but produced much less clearly defined patterns in the centre. Using a double spacer with a cell height of $\sim 160\,\mu$m lead to the onset of thick fingers that were kinetically hindered to form the transition to finer fingers at the centers, but we still observe monolayer deposition as before. Adding the polymeric triblock surfactant Pluronic® F127 suppressed finger formation completely. In Fig. S5, we also show that we observed the same pattern formation phenomenon with a rectangular geometry.

Rate of evaporation

In the experiments, the detached droplet in Fig. 4 dries in about 5 days from an initial radius of 5mm. The average observed drying speed is then of order 10^{-6} cm s^{-1}, which is much slower than the estimated shrinking speed that the same droplet would have if it were in contact with dry air ($\sim 2.7 \times 10^{-5}$ cm s^{-1}).

We can estimate how quickly a cylindrical droplet of the same size would evaporate into dry air at 25° C. The steady-state concentration profile around a cylindrical droplet with radius R in a cell with a radius R_0 depends logarithmically on the distance r from the center of the droplet:

$$\rho(r) = \rho(R) + \frac{\ln r/R}{\ln R_0/R}(\rho(R_0) - \rho(R)),$$

where $\rho(r)$ denotes the number density of the water vapor at radius r. We assume that the density of the water vapor at the boundary of the droplet is that of the saturated vapor $\rho(R) = \rho_{\mathrm{sat}}$, where ρ_{sat} is the saturated water vapor pressure at ambient conditions. If the cylindrical droplet is evaporating into almost dry air, we can assume that the transport of water vapor is diffusion-controlled. The radial concentration gradient is then:

$$\nabla c(r) = -\rho_{\mathrm{sat}} \frac{1 - R_H}{r \ln R_0/R}.$$

where R_H is the relative humidity of the air at the outer boundary of the cell. The total diffusive flux of water vapor per unit height J_D, is

$$J_D = \frac{2\pi D \rho_{\mathrm{sat}}(1 - R_H)}{\ln R_0/R}.$$

The diffusion coefficient of water in air at 25° C is approximately 0.24 cm^2 s^{-1}.As the droplet shrinks, R_0/R is not constant, but to obtain an estimate, we take a typical value: $R = 0.5R_0$. The flux of water (per unit length) that must evaporate due to the shrinkage of the droplet equal to

$$J_s = -\rho_L \pi \frac{dr^2}{dt}.$$

Balancing the two fluxes, we get:

$$\frac{2D\rho_{\mathrm{sat}}(1 - R_H)}{r \ln R_0/R} = 2\rho_L \dot{r}$$

The saturated vapor pressure of water vapor at 25° C is ≈ 0.032 atm. Hence, its molar volume is 7.8×10^5cm^3 (assuming an ideal gas molar volume of 24.46 l). Under the same conditions, the molar volume of liquid water is approximately 18 cm^3. Therefore, if $R_H = 0$ (dry air):

$$\dot{r} \approx \frac{D\rho_{\mathrm{sat}}/\rho_L}{r \ln R_0/R} \approx 2.710^{-5} \mathrm{cm\,s}^{-1}.$$

If the water is in contact with air with a relative humidity RH, then the rate of evaporation is reduced, and the shrinkage of the droplet slows down

$$\dot{r}_{\text{partial}} = (1 - RH)\dot{r}_{\text{sat}}.$$

As the experimentally observed shrinking speed is of the order $10^{-6}\,\mathrm{cm\,s^{-1}}$, we conclude that the relative humidity of the air at the outer rim of the cylindrical cell is approximately 96%.

As the atmosphere outside the cell has a typical relative humidity of ∼30-50% (Norway), it follows that largest drop of the chemical potential of the water is inside the scotch-tape seal. The implication is that the water evaporative flux is effectively constant until the droplet has disappeared.

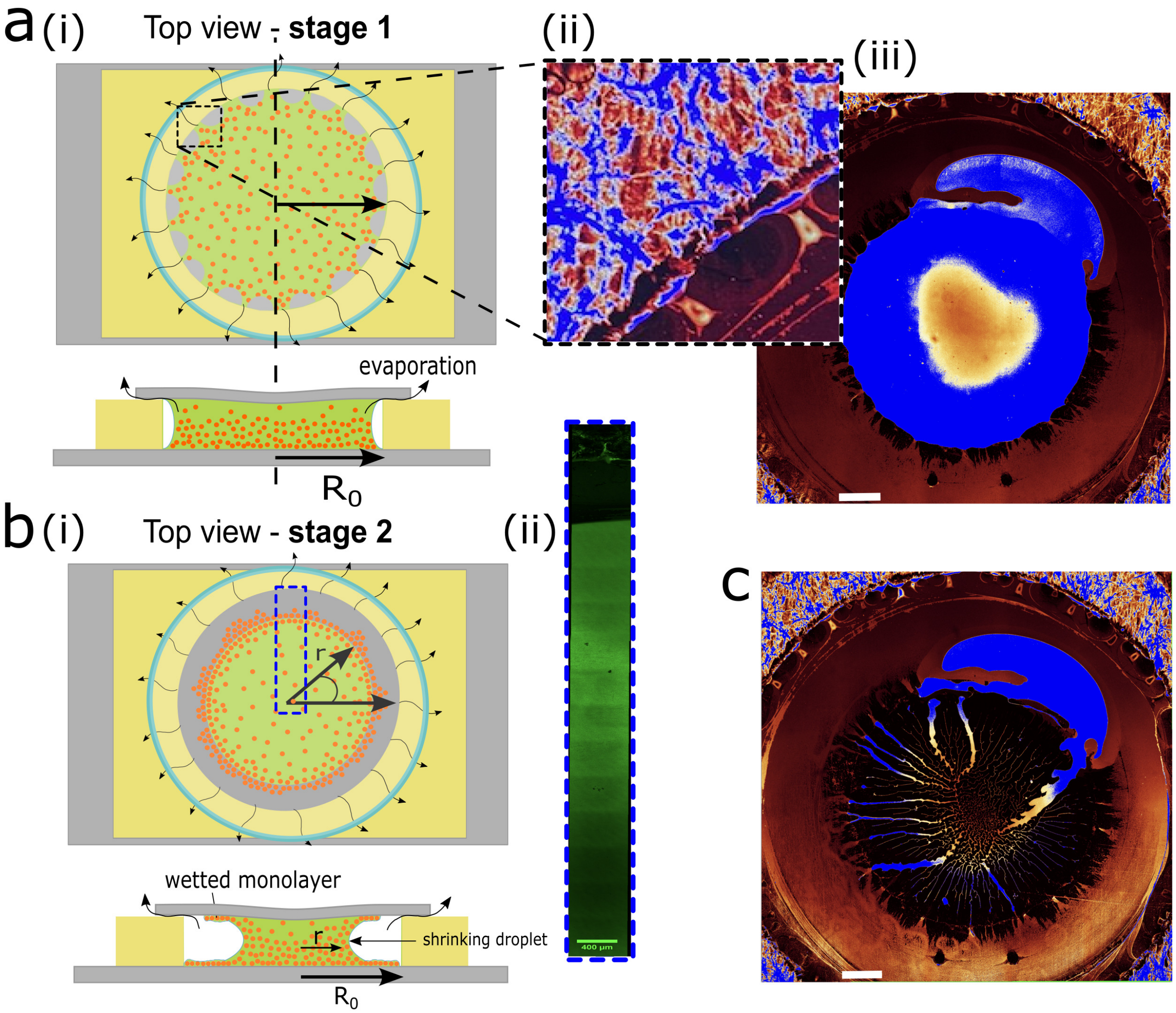

Fig. S1. Schematic and confocal images of a confined drying droplet containing 1.8 μm large TPM particles, and fluorescently dyed water, shown at different drying stages. **a** (i) Schematics of the top-down and cross-sectional view of the sample at **stage 1** of the drying process, showing the formation of air bubbles at the interface between the liquid and the double sided tape. (ii) Confocal image of the top surface of the cell that shows the fluorescent water trapped between the coverslip and double sided tape spacer. Blue indicates a high, and red a very low, water content. The almost black regions bounded by the red to yellow inside the bubbles are dry and almost void of colloids. (iii) 5×5 tiled confocal image show the entire sample cell (total image size 2560×2560 pixels), taken at the onset of **stage 3**. The scale bar is 1 mm. The schematics in **b**(i) illustrates the onset of monolayer deposition after the air bubbles have coalesced. The blue dashed rectangle corresponds to the composite confocal-image stacks in (ii), taken from the centre of the sample to the tape at the edge of the cell. The sharp black-to-green interface at the top, shows the outer air-suspension interface, while the integrated intensity decreases toward the centre of the sample, reflecting a dimple in the coverslip cause by capillary pressure. **c** A tiled confocal image of the sample (after 4 hours and 20 minutes relative to **a** (iii)) when the entire pattern has formed, but is still wet. See the full drying sequence in Video **V2**.

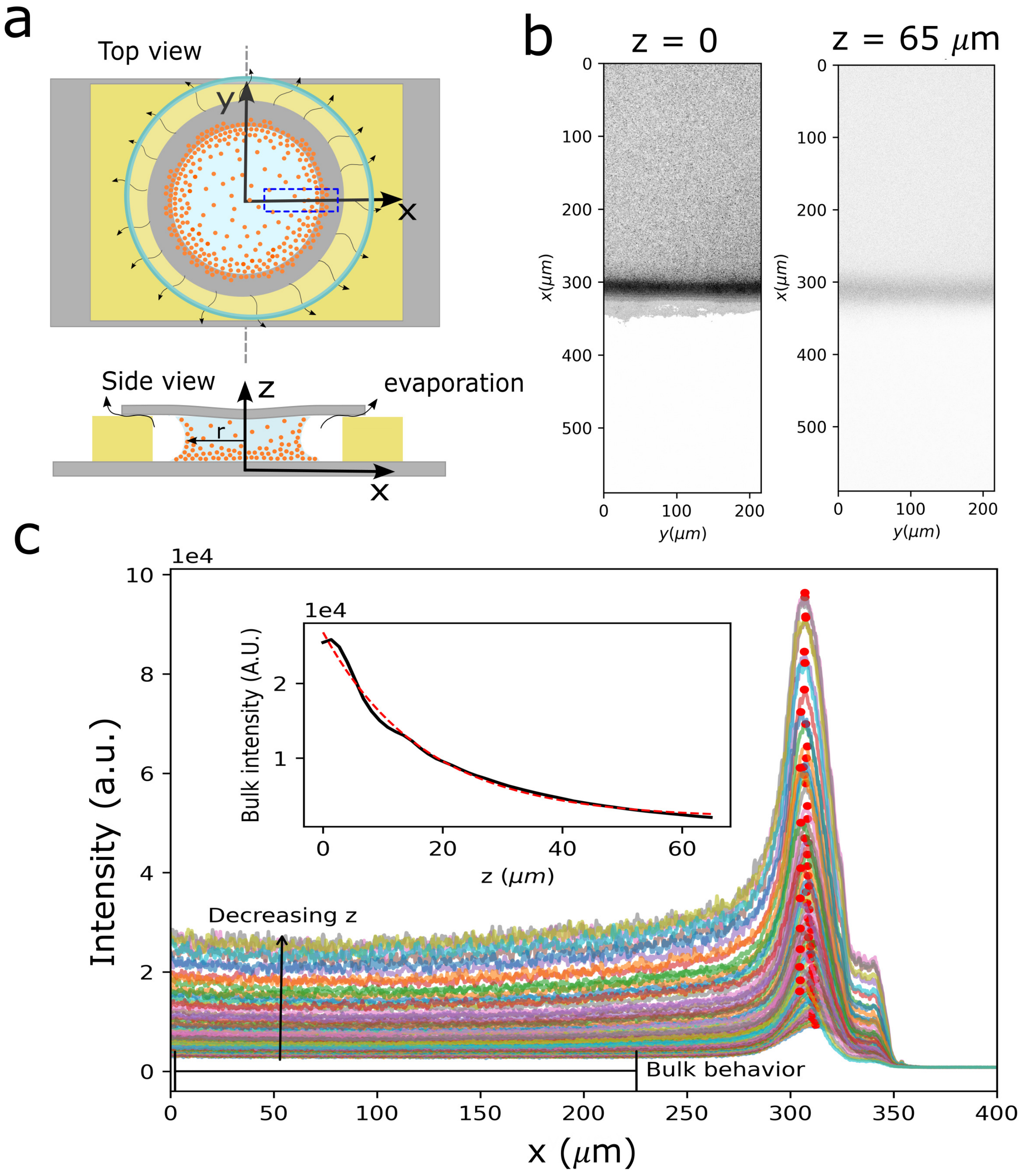

Fig. S2. Colloid distribution at the droplets' air-water interface. **a** Schematic of a droplet of the colloidal suspension placed in the middle of a sample cell, with both top-down and cross-sectional views. As this was placed in the middle, rather than allowed to dry to this stage on its own, monolayer deposition has only just begun at the droplet edge at this stage. The blue dotted rectangle corresponds to the area shown in the two confocal images in **b**. The two images are individual slices from a stack on confocal images through the entire thickness of the sample (approx. 80 μm). The image on the left hand side is taken at z = 0, corresponding to the bottom surface of the cell, while the image on the left is taken at z = 65 microns (approx. 15 μm below the top surface). **c** An intensity plot of all the images within the confocal image stack of the interface, stacked in the z direction from top to bottom. The inset plot shows the average bulk intensity from the bottom of the cell (z = 0) almost to the top of the cell at z = 65 μm.

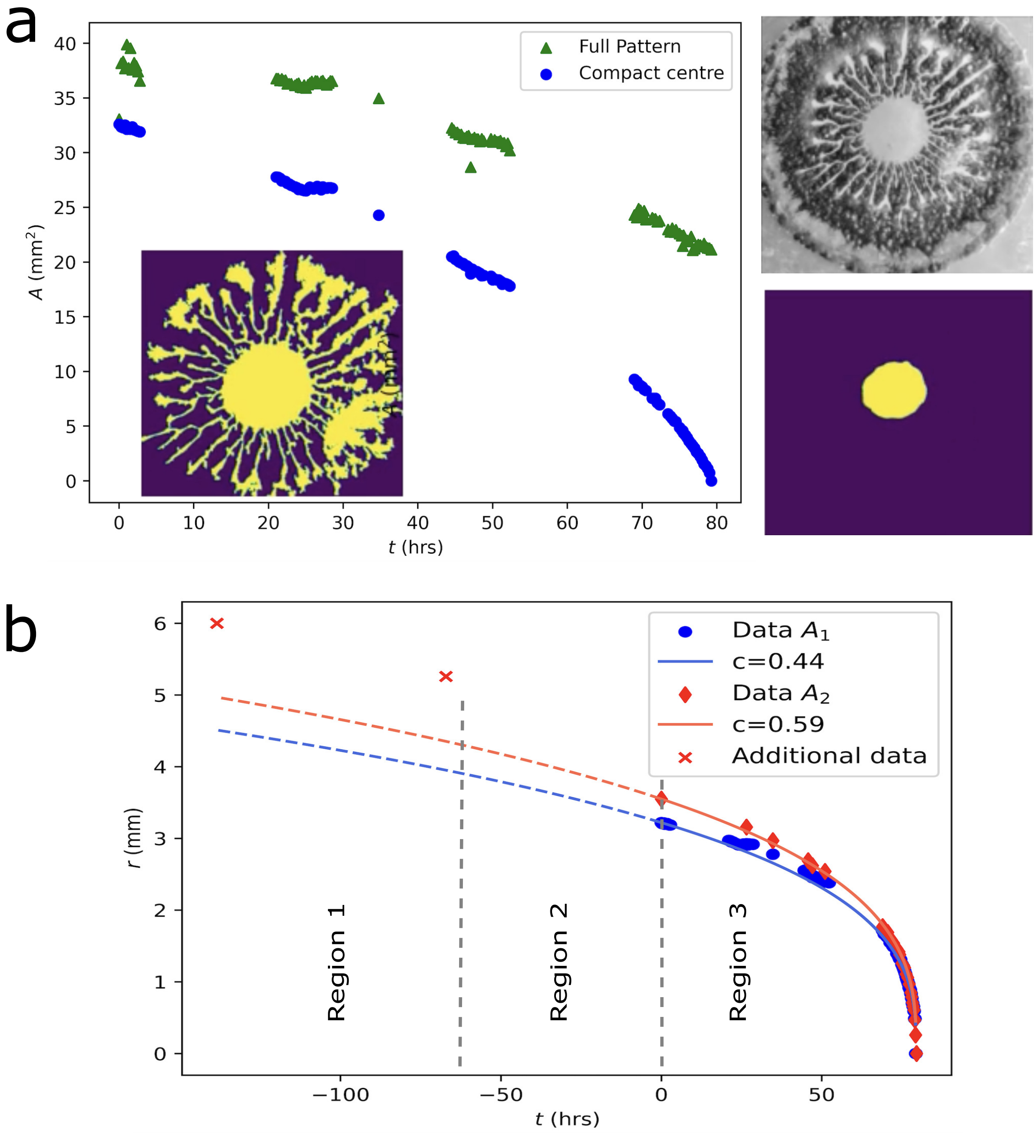

Fig. S3. Analysis of the drying kinetics shown in Fig. 3 in the main text. **a** Plot of the inscribed area A_1, which was estimated using method 1 (compact centre) and the area of the full pattern that includes also the area of the fingers. Snapshots of the respective thresholded patterns obtained for a given time at around $t = 70$ hours are shown, where the area is yellow and the background is purple. The corresponding original photograph is also shown. **b** Average radius of the inscribed circle extracted from the areas of the inscribed circles plotted in **a** and using method 2, which correspond to the data for $r(t)$ shown in Fig. 3 in the main text. The corresponding fitting lines are plotted as solid lines and continued to the start of the drying process at $t = -166$ hours as dashed lines. Region 1 corresponds to the bubble formation and coalescence, region 2 to the monolayer deposition and region 3 to the finger formation.

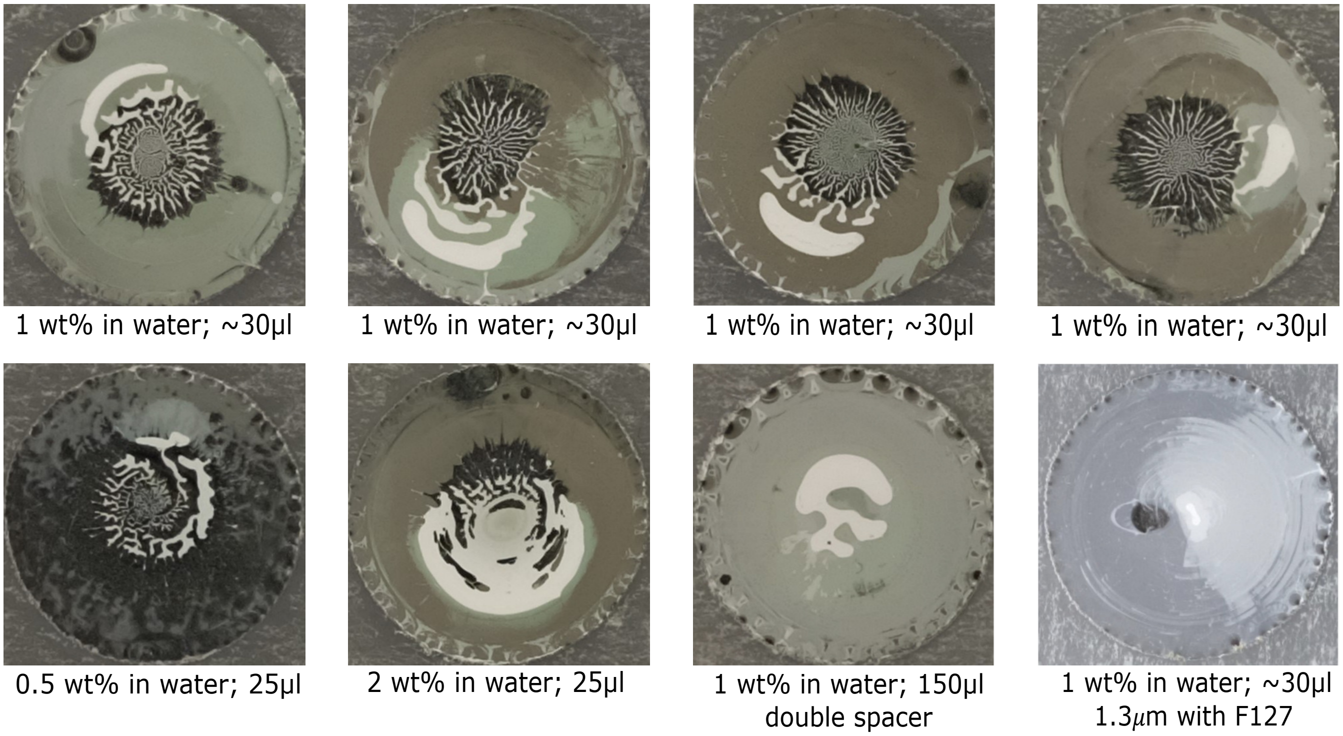

Fig. S4. Gallery of samples photographed in reflection. The top row shows 4 other samples with the same composition as those in the main text, showing high levels of reproducibility. The bottom row shows some other samples, each with a different modification. From left to right: 0.5wt% concentration of TPM, 2wt% concentration of TPM, double-spaced surfaces (cell height of 160 μm), and 1.3 μm large TPM colloids that have been surface functionalised with Pluronic® F127.

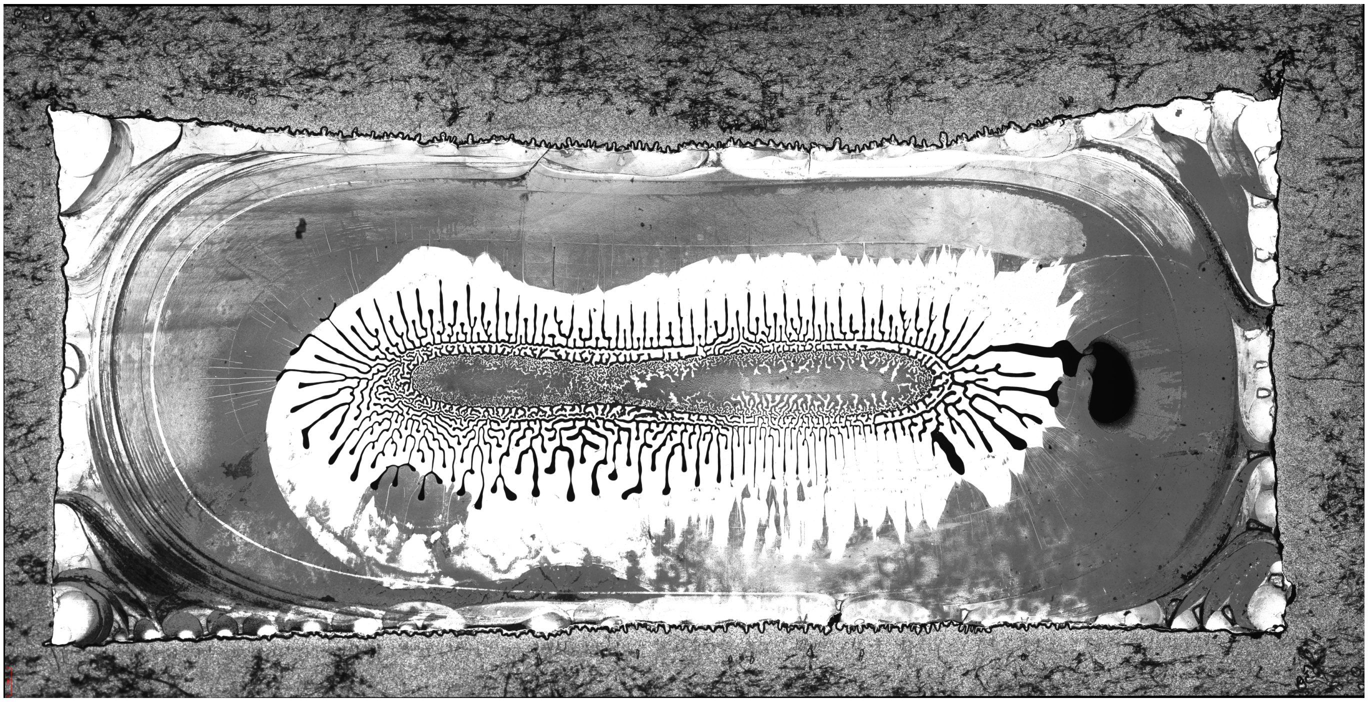

Fig. S5. Transition micrograph of a dried sample that was prepared with a suspension of 1 wt% of 1.8 μm large TPM particles. The image was reconstructed from a 12$\times$6 tiling of micrograpghs. The square cell is 24 $\times$ 12 mm large.

Movie S1. V1 - Time-lapse movie of drying process for sample presented in Figure 2a in the main text.

Movie S2. V2 - Time-lapse movie of finger formation process of sample presented in Figure S1.

Movie S3. V3 - Full stack of confocal images taken at the droplet interface presented in Figure S2.

Movie S4. V4 - Time-lapse movie of finger formation process for the analysis presented in Figure 4 in the main text, and Figure S3.

References

1. PG De Gennes, F Brochard-Wyart, D Quéré, *Capillarity and Wetting Phenomena.* (Springer New York, New York, NY), (2004).
2. Y Liu, et al., Colloidal organosilica spheres for three-dimensional confocal microscopy. *Langmuir* **35**, 7962–7969 (2019).
