# Supplementary figures and images for: Confined colloidal droplets dry to form circular mazes
Source: Proc Natl Acad Sci U S A. 2025 Aug 4;122(32):e2508363122. doi: 10.1073/pnas.2508363122 (PMC12358886; doi:10.1073/pnas.2508363122)

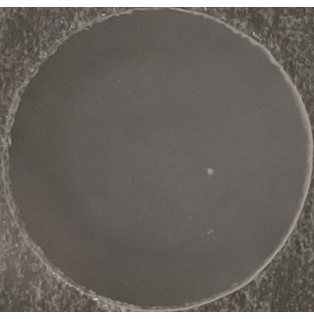

Supplement: Movie S1. — V1 - Time-lapse movie of drying process for sample presented in Figure 2a in the main text. [file pnas.2508363122.sm01.gif]

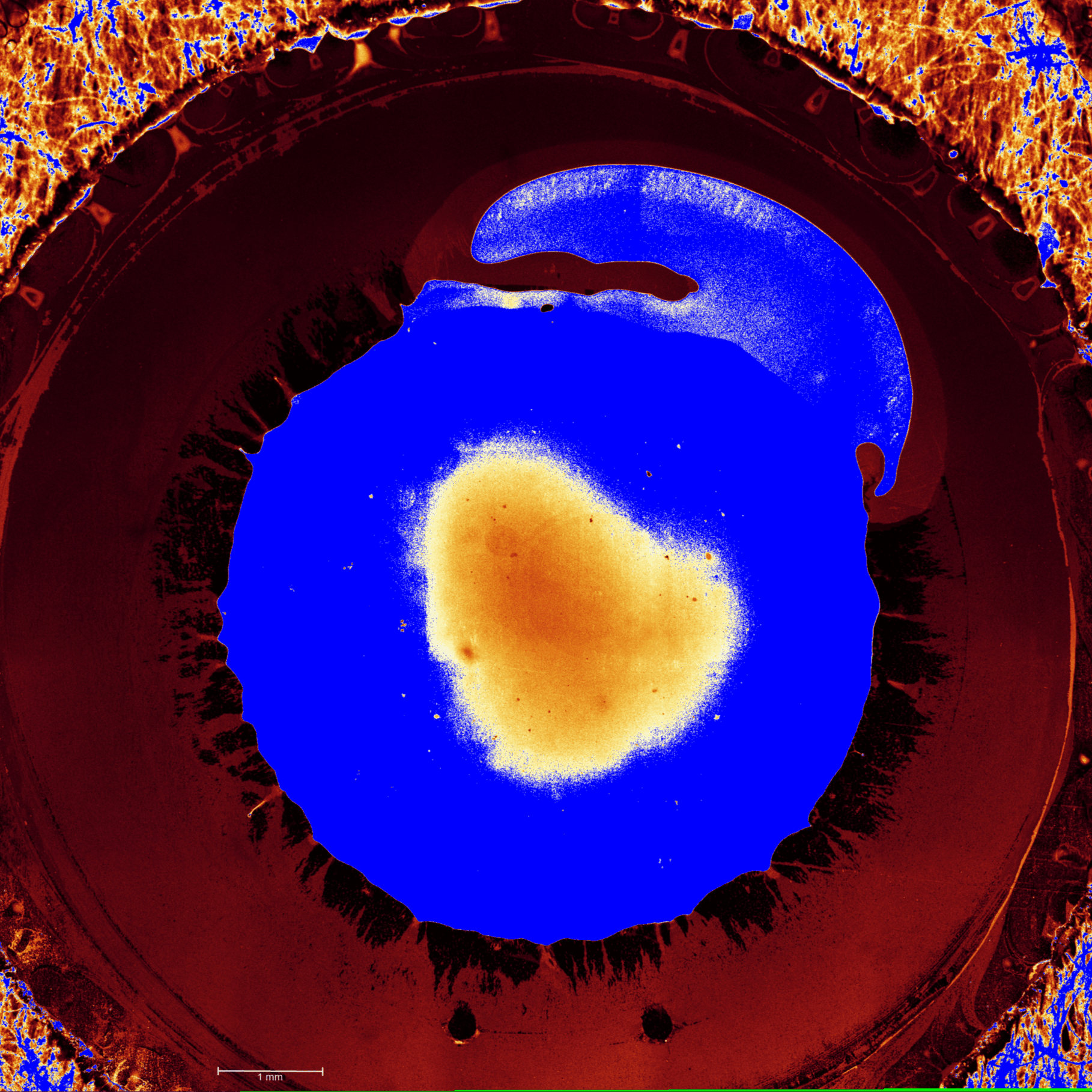

Supplement: Movie S2. — V2 - Time-lapse movie of finger formation process of sample presented in Figure S1. [file pnas.2508363122.sm02.gif]

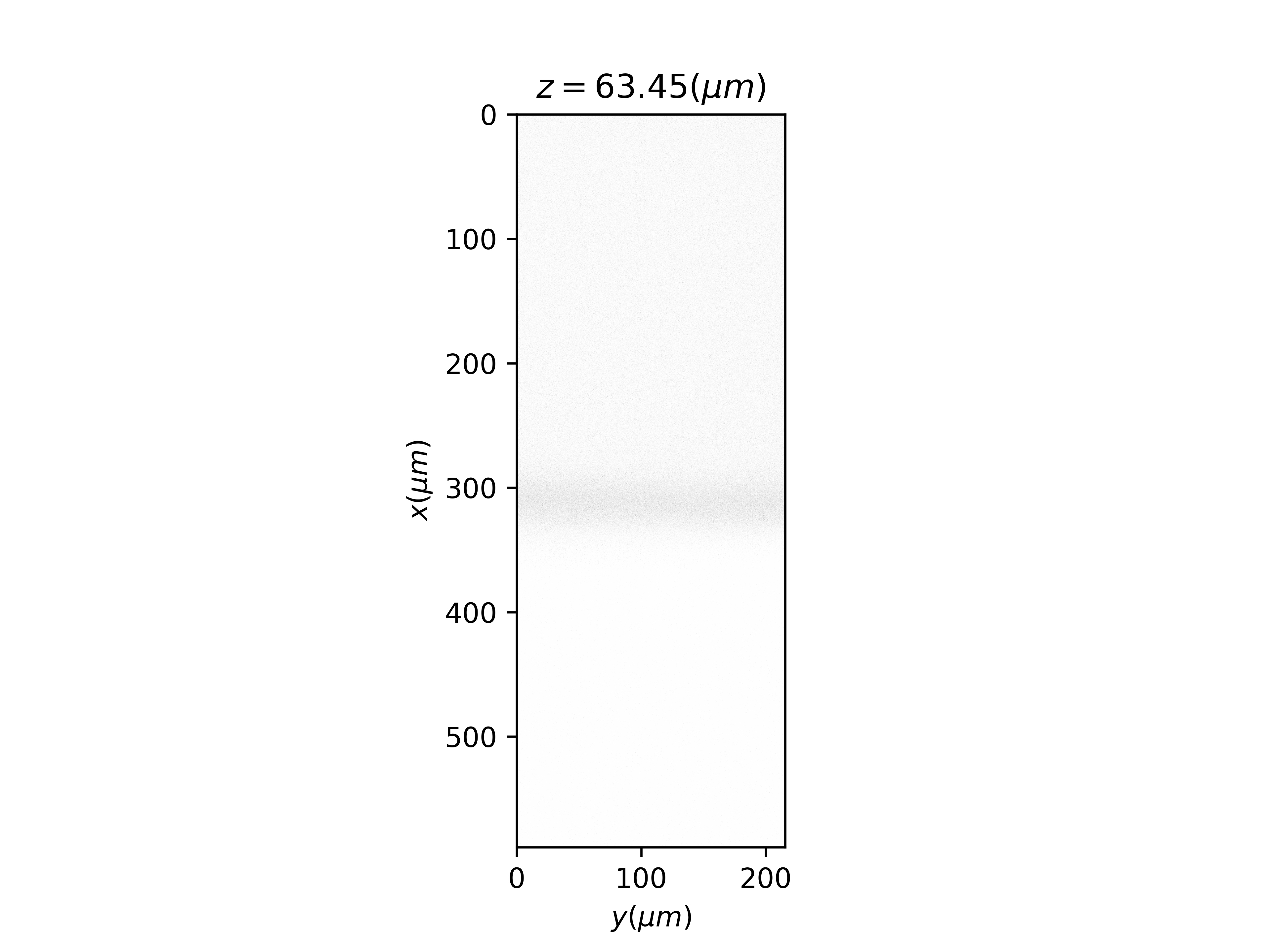

Supplement: Movie S3. — V3 - Full stack of confocal images taken at the droplet interface presented in Figure S2. [file pnas.2508363122.sm03.gif]

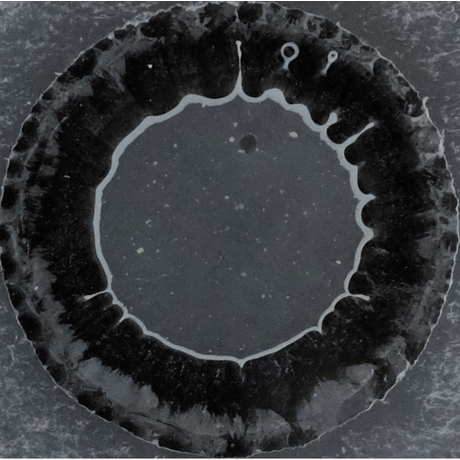

Supplement: Movie S4. — V4 - Time-lapse movie of finger formation process for the analysis presented in Figure 4 in the main text, and Figure S3. [file pnas.2508363122.sm04.gif]
